# On pandemics, pandemonium, and possibilities…

**DOI:** 10.1096/fba.2020-00023

**Published:** 2020-05-12

**Authors:** Ralph A. Bradshaw, Philip D. Stahl

**Affiliations:** ^1^ FASEB BioAdvances

We are now over 3 months into a viral **pandemic** (COVID 19) that has already left a trail of death and destruction, wreaking social and economic havoc, and disrupting, in most parts of the world, just about every aspect of life—and it is far from over. It is therefore a source of some pride that the scientific and medical professions have responded in exemplary fashion and continue to do so. Despite the interruptions to their research programs and careers, scientists have pooled resources and expertise, and health care providers have manned the trenches. The levels of cooperation and sharing throughout the global community are unprecedented as medicine and science have collectively taken on a common foe, often accompanied by the refocusing of individual work at the expense of established activities. There are multiple efforts afoot to develop a vaccine; a number of antivirals are being tested, as are pharmacopoeias of approved drugs that might be repurposed for treating COVID 19; and new drug development using the powerful tools of modern cell and molecular biology have been launched. Indeed, medicine and the biomedical research community has much to show for itself already, and these efforts have certainly justified the generous public support of medical research over the years.

This is an important message that needs to be heard on Capitol Hill, and it is a great time, particularly with work and travel restrictions still largely in place, to contact representatives and senators and remind them that there is no better dollar spent by the government than that which supports biomedical research. The **possibilities** that could emerge after the pandemic has wound down are truly unique—a potential watershed moment for science, sparked by a new focus on science teaching and training, and by investments in basic and applied research (including science policy), that could better prepare governments and the citizenry for existential threats in the future: pandemics, climate change, food production, etc. Continued investments in biomedical research will also continue to yield exciting advances in the treatment of other killers such as cancer, heart disease, and neurodegeneration that, unlike the pandemic, will not have faded away.

Indeed, there are likely to be many permanent changes in how we live and work that will emerge from the **pandemonium** (The chaos all around us may not be pandemonium in the usual sense—although Merriam‐Webster does define pandemonium as “a chaotic situation”—but when one considers the economic/social disruptions that have already occurred, it does not seem like such a bad description). For example, there have been significant (positive) effects on air quality resulting from the dramatic reductions in local, domestic, and international travel, and it is likely that telecommuting will become a much more attractive proposition for companies and businesses that are able to incorporate such programs into their operations. Similarly, web‐based teaching will almost certainly become more prevalent, and there are apt to be substantial changes in how higher education is managed by colleges and universities.

Although only a couple of months ago most people did not even know the word, pandemics are not new. In fact, we are only just past the 100th anniversary (1918‐19) of the worst (modern) pandemic, commonly known as the Spanish Flu, caused by the influenza A (H1N1) virus.^1^ Since there remains essentially no living memory, the populace today are not really aware of the extent and consequences of this scourge: estimated number of people infected: ~500 million (or about one‐third of the world's population at that time) and estimated number of deaths: 50 million worldwide (~675 000 occurring in the United States, or about 0.6% of the US population of about 100 million at that time). If one‐third of today's world population eventually contracted COVID 19, it would affect ~ 2.5 billion people and, at the presently calculated level of lethality, cause over 150 million deaths. There have also been three other influenza pandemics that occurred in 1957‐58, 1968, and 2009, each triggered by a new influenza virus variant. None of these were as deadly as the 1919 agent, but in the first two instances, there were still over 1 million deaths.^1^

Of course, the influenza A (H1N1) and SARS‐CoV‐2 viruses and the diseases they cause are not directly comparable nor are the circumstances in which the pandemics occurred. While the management of the 1918‐19 flu was hampered by a complete lack of knowledge of the causative agent or how to test for it,^1^ the identification and a complete structural analyses of the SARS‐CoV‐2 coronavirus^2^ occurred very rapidly after the onset of the pandemic in December 2019, and assays of different types for it were developed in just weeks,^3^ albeit making these tests widely available has been a major challenge (but this is a political not a medical problem). This is a very significant difference since they do share two key aspects: the causative viruses are unique, that is, no one was exposed to either before, and there was and is (presently) no effective treatment for either affliction.^1^ Both then and now, prophylaxis that limits or prevents person‐to‐person transmission was and still is the only effective means for stemming the spread of the pandemic with all the limits and impact on society that that approach must confront. Therefore, being able, at least in theory, to identify (and potentially track) individuals who test positive is an enormous advantage that health care providers in 1918 did not have; we have the great strides of bioscience to thank for this. The H1N1 flu occurred in three waves in the United States over a period of a little greater than a year,^1^ first appearing in April 1918 and ending in the summer of 1919. Whether COVID 19 will follow a similar course is not at the moment predictable.

While there is clear evidence that “social distancing” combined with “shelter at home” and the elimination of all social events characterized by any‐sized groups of unrelated people has helped to flatten the curve of new infections, it has also come with severe consequences. Unhappily, it is not hard to conclude that at least a part of the problems introduced may have arisen from the unintended consequences that often accompany precipitous, hasty, and not well thought through decisions. Indeed, worldwide leadership from national to local levels has not been uniformly inspiring, being often slow to act and then overreaching. Postulations about possible treatments/approaches based on wishful thinking are not a substitute for scientific facts. It is particularly disturbing to realize that scientists, health care providers, and related experts had anticipated the eventuality of another pandemic. They issued warnings and prepared detailed management plans for just such an event^1^ that were to a large degree unheeded. It may have not been possible with the tools and knowledge available a hundred years ago to have significantly curtailed the Spanish Flu, but the lessons learned from that experience provided excellent guidance of how to react in the future, and that was sadly largely ignored till the magnitude of the COVID 19 outbreak became unavoidably clear.

Like earthquakes, one does not know when the next pandemic will occur, only that it will, and that when it does, science and medicine will be called upon to address it. We need to be better prepared.

Call your congressional representatives and remind them of that.
